# Supplementary material for: Emergence of Collective Memories
Source: PLoS One. 2010 Sep 1;5(9):e12522. doi: 10.1371/journal.pone.0012522 (PMC2931705; doi:10.1371/journal.pone.0012522)

Supporting Information

In this Supporting Information we present a picture showing the stability of Fig. 5A to the misunderstanding parameter ρ. We use ϕ = 0.3 and keep the other parameter values the same as in Fig. 5. The error bars are smaller than symbol size. If there is misunderstanding, or noise, in the system the maximum number of groups is lower and the peak is split in two, the larger peak happens at larger *r*-values. The main quantitative conclusion from the study at ρ = 0, the existence of a diversity regime, remains. The split peak, we believe, is an effect of the communities nucleating more heterogeneously. Around *r* = 500 there are some stronger communities that takes more of the links than in the noiseless case, whereas other groups, that would be communities without noise, the gets grouped into one. The effect of this would, we believe, be the observed dip in the $g(\rho)$-curve around *r* = 500. Yet increasing communication makes the system able to overcome the noise so that the peak is almost recovered although displaced toward larger ρ.

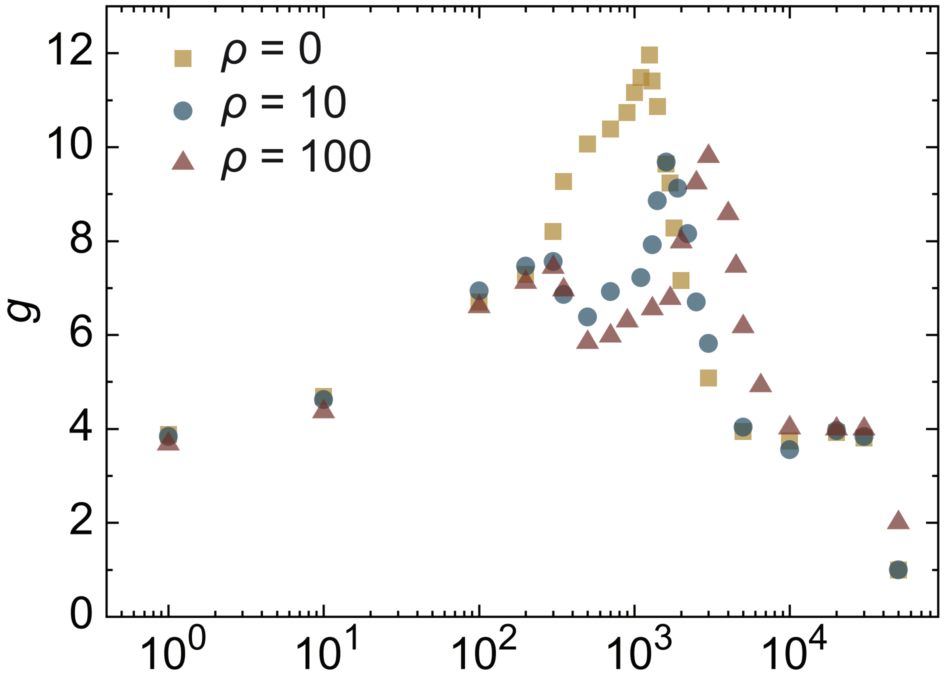

Supplement: Supporting Information S1 — Supporting Information investigating the stability of Fig. 5A. (0.16 MB PDF) [file pone.0012522.s001.pdf]
